# Structures, properties, and energy-storage mechanisms of the semi-lunar process cuticles in locusts

**DOI:** 10.1038/srep35219

**Published:** 2016-10-17

**Authors:** Chao Wan, Zhixiu Hao, Xiqiao Feng

**Affiliations:** 1 Institute of Biomechanics and Medical Engineering, Department of Engineering Mechanics, Tsinghua University, Beijing 100084, China; 2 State Key Laboratory of Tribology, Tsinghua University, Beijing 100084, China

## Abstract

Locusts have excellent jumping and kicking abilities to survive in nature, which are achieved through the energy storage and release processes occurring in cuticles, especially in the semi-lunar processes (SLP) at the femorotibial joints. As yet, however, the strain energy-storage mechanisms of the SLP cuticles remain unclear. To decode this mystery, we investigated the microstructure, material composition, and mechanical properties of the SLP cuticle and its remarkable strain energy-storage mechanisms for jumping and kicking. It is found that the SLP cuticle of adult *Locusta migratoria manilensis* consists of five main parts that exhibit different microstructural features, material compositions, mechanical properties, and biological functions in storing strain energy. The mechanical properties of these five components are all transversely isotropic and strongly depend on their water contents. Finite element simulations indicate that the two parts of the core region of the SLP cuticle likely make significant contributions to its outstanding strain energy-storage ability. This work deepens our understanding of the locomotion behaviors and superior energy-storage mechanisms of insects such as locusts and is helpful for the design and fabrication of strain energy-storage devices.

Jumping and kicking are typical activities of locusts and crucial for their survival in nature. Locusts jump for escaping from predators, launching themselves for flight, and travelling more quickly than walking. Their jump performance is excellent with high velocity (3.2 m/s), rapid acceleration (180 m/s^2^), and long distance (more than ten times the body length in each jump)^1^. Kicking helps locusts repel predators or conspecifics. During kicking, the hind legs move even more rapidly and powerfully^2^. Both the jumping and kicking activities are achieved by the rapid extension of the hind tibia, which is mainly actuated by the extensor tibial muscles around each metathoracic femorotibial joint^3^. However, the immediate contraction of muscles cannot provide sufficient energy needed by the rapid jumping and kicking because these processes happen in a very short time (25–30 ms for jumping and approximately 6 ms for kicking)^1^,^2^,^4^,^5^. The power output of the muscle’s contraction during these short times is far below the total energy required by each motion.

It has been recognized that a large amount of strain energy is stored by some deformed exoskeletal cuticles, especially in the semi-lunar process (SLP), before the jumping of a locust^6^. The SLP is a banana-shaped, highly sclerotized, thickened cuticle on the distal end of the metathoracic femur of locusts^1^ (shown in Fig. 1a,b). During jumping, the SLPs rapidly (25 to 30 ms) recover to their undeformed configuration and release the stored elastic strain energy^1^. The rapid energy release induces the legs to extend under high force and at high velocity^4^,^7^. A similar pattern of strain energy storage is also adopted by locusts for kicking. High-speed camera images have showed that a greater deformity occurs in the SLP before kicking than jumping, signifying more strain energy storage required for kicking^1^,^2^,^8^.

Due to its significant role in locomotion, the SLP has attracted considerable attentions in recent years^1^,^8^,^9^. Gabriel reported anatomical changes in the SLPs and found that SLP thickness increased as locusts matured^9^. Ultraviolet images revealed that the SLP comprises a hard cuticle outside and rubber-like protein, resilin, inside^8^. The sclerotized cuticles store most of the strain energy because of their higher elastic modulus, whereas resilin tissues contribute to preventing cuticle fractures^8^,^10^,^11^. The microstructures, material compositions, and mechanical properties of some other cuticles of adult locusts have also been investigated, including those in sternal plates and tibiae^8^,^10^,^12^,^13^,^14^. It was found that the cuticles on sternal plates have distinct multilayer microstructures with anisotropic mechanical properties, which are closely related with water content^12^. However, there is lack of research on the microstructure, material composition, and mechanical properties of the SLP cuticles, and their strain energy-storage mechanisms remain elusive. Whether differences exist between the strain energy-storage mechanisms for jumping and kicking also have not been identified.

In this work, we investigated, experimentally and theoretically, the microstructure, material composition, and mechanical properties of the SLP cuticles of adult *Locusta migratoria manilensis*. Finite element (FE) simulations of distal femurs (including the SLP cuticles) were performed to elucidate their strain energy-storage mechanisms of the SLP cuticles for the jumping and kicking of locusts. We will explore the microstructure-composition-property-functions relations of the different structural components in SLP cuticles, and their deformation and energy-storage mechanisms for jumping and kicking.

## Results

### Microstructural characterization

**Figure 1:**
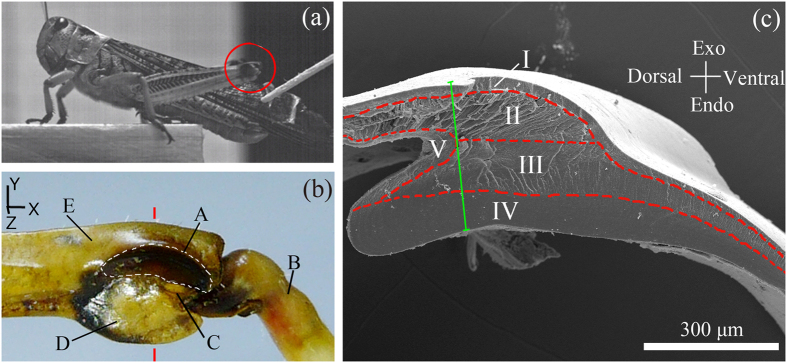
Anatomy of the femorotibial joint of the hind leg of an adult *Locusta migratoria manilensis* and the cross-sectional view of its semi-lunar process (SLP). (**a**) An adult *Locusta migratoria manilensis*. Its femorotibial joint of hind leg (marked by a red circle) is at full flexion. (**b**) The femorotibial joint is viewed with a high magnification, where A, B, C, D, and E represent the SLP, tibia, soft membrane, cover plate, and femur, respectively. The SLP cuticle is marked by white dashed lines along its boundary. A right-handed coordinate system is established for the joint, where the *X* axis is along the longitudinal direction (i.e., distal direction) of the femur, *Y* in the tangential direction (i.e., dorsal direction) of the exoskeletal shell, and *Z*-axis in the normal direction (i.e., lateral direction) of the exoskeleton. (**c**) Scanning electron microscopy (SEM) image for the cross-section of a unilateral SLP cuticle in the femorotibial joint. The five portions in the SLP cuticle are distinguished by the red dashed lines along their interfaces. The thickest cuticle in the SLP cross-section, marked by the green solid line, has four portions, I–IV. The SLP cuticle was sectioned along the red solid line shown in (**b**).

**Figure 2:**
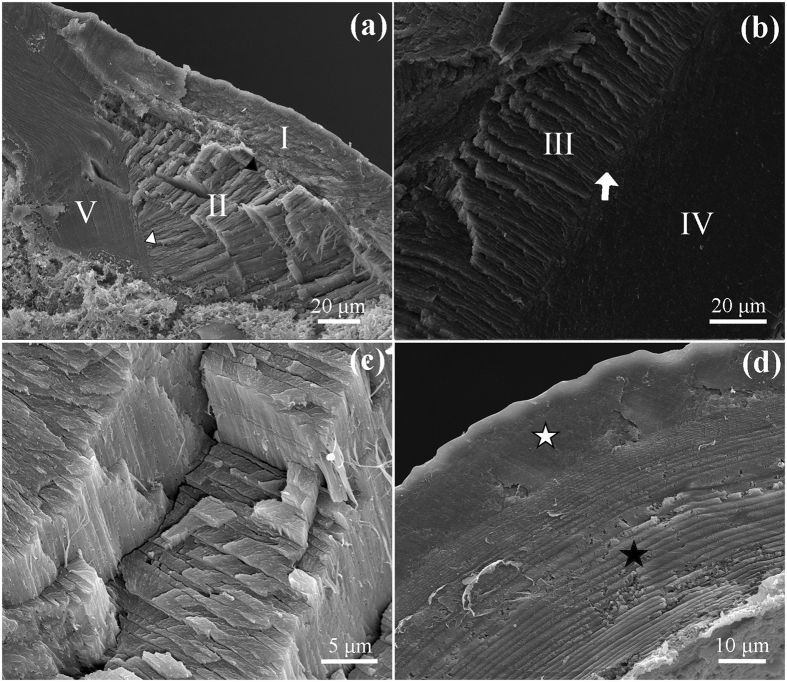
SEM images of the five portions of the SLP cuticle. (**a**) The cross-sectional image of the dorsal part of the SLP cuticle. The interfaces between portions I and II and between portions II and V are marked by solid and empty triangles, respectively. (**b**) The microstructures of portions III and IV. Their interface is marked by the white arrow. (**c**) Microstructure of portion II, which has regularly distributed and aligned microfibers along the longitudinal direction of the femur. (**d**) Cuticle samples from the dorsal-distal exoskeleton of the femur, which has a solid shell outside (~20 μm in thickness, marked by an empty star) and a multilayer structured core (~40 μm, marked by a solid star).

Scanning electron microscopy (SEM) images of six SLP specimens reveal that the SLP cuticles have five structural parts that are bonded together with clear interfaces, which are marked as I–V in Fig. 1c. The different parts possess with distinctly different microstructures. Regular microfibers exist in the dorsal part of the core (portion II in Fig. 2a), whereas the microfibers in the ventral part of the core are irregular in shape and in direction (portion III in Fig. 2b). As can be seen from the high-magnification SEM image in Fig. 2c, the microfibers in portion II are regularly distributed and aligned almost along the longitudinal direction of the femur (the *X*-axis in Fig. 1b). The other parts of the SLP cuticles also have different microstructures. The exo-surface layer (portion I in Fig. 2a) is a thin shell of ~20 μm in thickness, the endo-dorsal part near the SLP (portion V in Fig. 2a) has a multilayer structure, and the endo-ventral part (portion IV in Fig. 2b) has a dense solid substance. For comparison, cuticle samples from the dorsal-distal exoskeleton of the femur were also observed, as shown by the SEM images in Fig. 2d. These cuticles mainly comprise a thin shell of ~20 μm in thickness and a core of multilayer structure of ~40 μm in size, which are similar to portions I and V of the SLP cuticles, respectively. The main difference between the microstructures of the two above kinds of cuticles lies in the microfibers in the core region of the SLP cuticles (including both portions II and III).

The characteristic sizes of the five SLP portions were measured and averaged from cross-sectional SEM images of six whole SLPs of adult *Locusta migratoria manilensis*. To eliminate the effect of data dispersity, we here give the relative dimensions of the five portions with respect to the whole size of the SLP cuticle. The thickest cuticle in the SLP cross-section is divided into four portions, i.e., referred to as I–IV, respectively, as shown by the green solid line in Fig. 1c. The relative thicknesses of portions I–IV with respect to the maximal thickness are about 7.4 ± 1.1%, 36 ± 1.1%, 34 ± 1.2%, and 22 ± 2.0%, respectively. The thickness of portion V is about 1.12 times that of portion I.

### Material compositions

**Figure 3:**
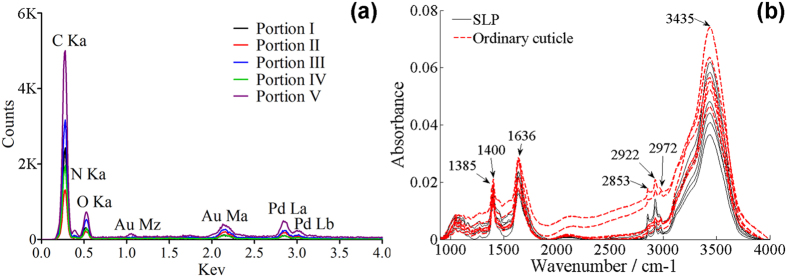
Element analysis and Fourier transform infrared (FTIR) spectrum of the SLP cuticle. (**a**) The energy dispersive spectroscopy (EDS) results of a typical SLP specimen are shown, which illustrate that no mineral element is found in any of the five SLP portions (the gold and palladium elements are introduced from the sputtered coatings for SEM). (**b**) The FTIR results of six SLP cuticle samples (black solid curves) further reveal that the functional groups in the SLP are similar to those in the cuticles of the dorsal-distal exoskeleton of the femur (red dashed curves).

The material compositions of the SLP cuticles were analyzed using energy dispersive spectroscopy (EDS), Fourier transform infrared spectrometry (FTIR), and confocal laser scanning microscopy (CLSM). The EDS results of six SLP specimens show that few kinds of mineral elements exist in all portions of the SLP cuticles (Fig. 3a). Among them, the gold and palladium elements in the spectrums originate from the sputtered coating of the samples for SEM. The FTIR results (n = 6) further reveal that the functional groups in the SLP cuticles are almost the same as those in the cuticles from the distal-dorsal part of femur (Fig. 3b). The absorbance curves have seven peaks in the functional group region (1300–4000 cm^−1^), including 1385 cm^−1^, 1400 cm^−1^, 1636 cm^−1^, 2853 cm^−1^, 2922 cm^−1^, 2972 cm^−1^, and 3435 cm^−1^.

**Figure 4:**
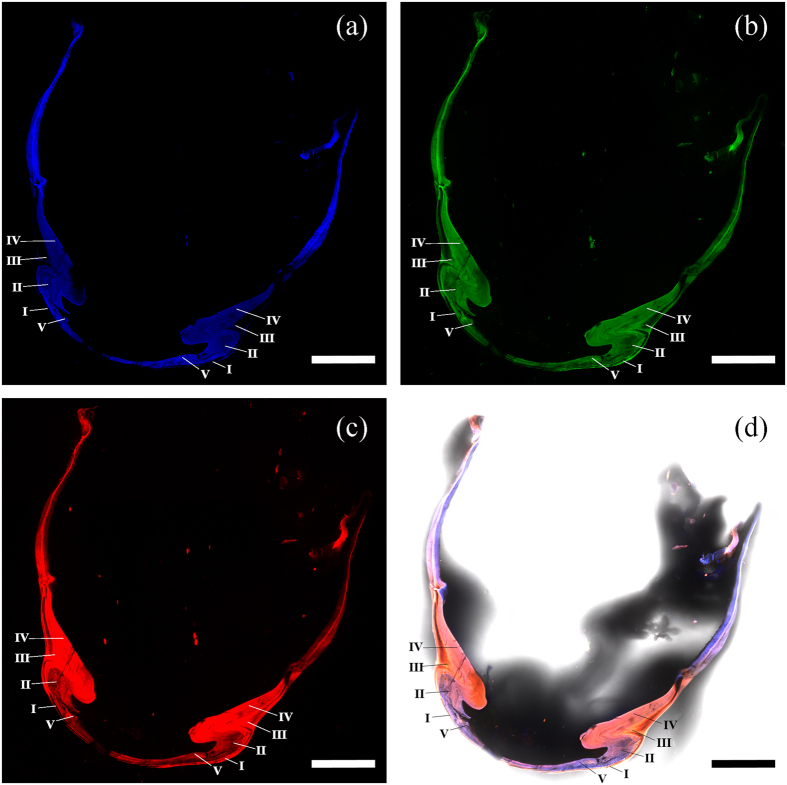
Maximum intensity projection images of a typical SLP cross-sectional specimen using CLSM method. (**a**) The blue autofluroscence (excitation = 405 nm and emission = 420–480 nm); (**b**) the green autofluroscence (excitation = 488 nm and emission ≥ 490 nm); (**c**) the red autofluroscence (for both: excitation = 555 nm and emission ≥ 560 nm; excitation = 639 nm and emission ≥ 640 nm). (**d**) All the different autofluroscences overlaid with the bright-field image of the cross-sectional SLP specimen. The different portions are labeled in the subfigures. Scale bars = 500 μm.

Six SLP samples were further used for CLSM analysis. The results reveal the different distributions of chitin and resilin in the five portions of each sample (Fig. 4). The red autofluorescence of portions I and III indicate that they are mainly composed of sclerotized cuticles (i.e., chitin). The autofluorescence of portion IV appears pink, implying that it consists mainly of chitin and a smaller amount of resilin. Portions II and V show a mixture of red and blue autofluorescences, and therefore they are composed of chitin and a higher content of resilin. The different colors result from the overlay of different autofluorescences, depending on the relative proportions of chitin and resilin. Some parts consisting of pure resilin (i.e., the inner surfaces of both the lateral and medial cover plates) show blue autofluorescence.

### Mechanical properties

**Figure 5:**
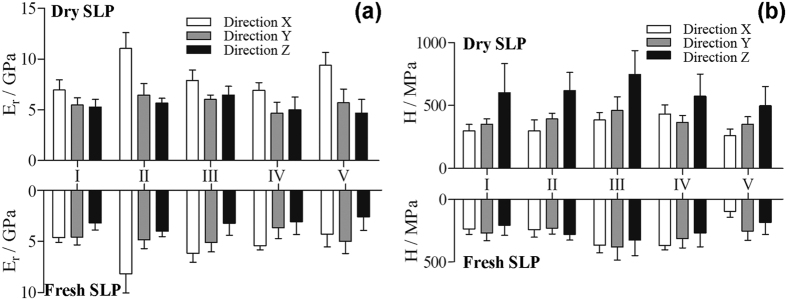
Mechanical properties of the five portions of the dry and fresh SLP cuticles. The reduced elastic modulus (**a**) and hardness (**b**) of the five portions along each direction were obtained from nanoindentation tests. The top and bottom diagrams correspond to the dry and fresh SLP cuticles, respectively. The directions are defined as those in Fig. 1b.

Nanoindentation tests were made to measure the mechanical properties of the five portions in each dry SLP cuticle. To determine the anisotropy of their properties, we measured all the length (*X*-axis), width (*Y*-axis), and thickness (*Z*-axis) directions of the SLP samples (six samples for each direction). The three axes are marked in Fig. 1b. In total, 270 indents were made on the eighteen specimens. It is found that the reduced elastic modulus *E*_*r*_ along the longitudinal (*X*-axis) direction, 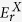, is significantly higher than those in the other two directions (p < 0.005, one-way ANOVA, Bonferroni post hoc test), as shown in Fig. 5a. The hardness *H* in the normal (*Z*-axis) direction, *H*^*Z*^, is also significantly higher than those in the other two directions (Fig. 5b). Additionally, the *E*_*r*_ and *H* in all five portions are similar in the width (*Y*-axis) and thickness (*Z*-axis) directions. The above results demonstrate that the dry SLP cuticles manifest transversely isotropic properties.

In addition, the heterogeneity of the mechanical properties in the dry SLP cuticles was further analyzed by comparing the mechanical properties of the five portions along the same direction (one-way ANOVA, Bonferroni post hoc test). The comparison of the 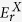 values shows significant differences (p < 0.005) in all comparisons of the five portions except between portions I and III, I and IV, and III and IV. Significant differences between the 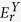 and 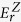 values of the five portions are found only between portions II and IV and between portions III and V, respectively. By contrast, the five portions of the dry SLP cuticles have almost the same hardness along each direction.

The mechanical properties of the five portions of fresh SLP cuticles were obtained from the above specimens after rewetted in distilled water because rewetted locust cuticles are demonstrated to exhibit the same mechanical properties as fresh cuticles^12^. There were also 270 indents performed on the eighteen specimens for the three directions. The mechanical properties of the five portions in the dry and fresh SLP cuticles were compared in order to determine the effect of water content. Regardless of portion and direction, the *E*_*r*_ and *H* values of the fresh SLP cuticles are all significantly lower than those of the dry SLP samples (t-test, P < 0.005). The 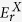 value of portion II is the highest among all the *E*_*r*_ values of the fresh SLP portions, followed by the 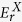 value of portion III. All the portions of the fresh SLP cuticles are transversely isotropic, but the isotropic plane of portions I, III, and V change from the transverse plane (*Y-Z* plane) to the sagittal plane (*X-Y* plane). By contrast, the *H* results of all the fresh SLP portions are similar, except that the *H* value of portion V along the *X-*axis, *H*^*X*^, is distinctly lower than those along the other two axes.

### Strain energy-storage mechanisms

**Figure 6:**
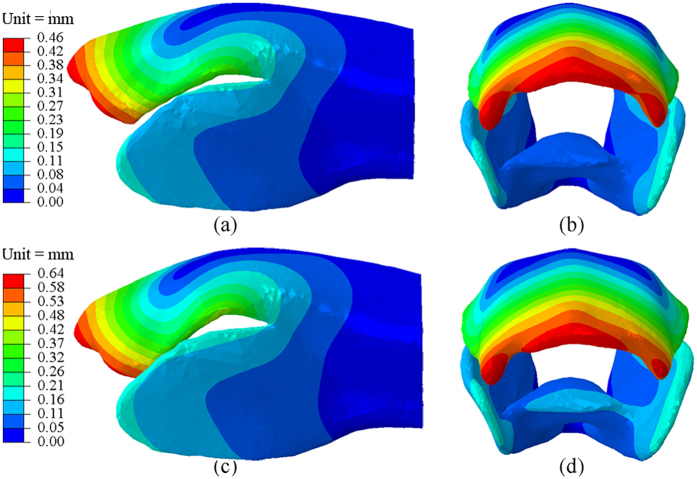
Finite element (FE) results for the deformations of the distal femur during the strain energy storages for jumping and kicking. The deformations are shown from the lateral and distal views in (**a,b**) for jumping and in (**c,d**) for kicking, respectively.

The strain energy-storage mechanisms of the SLP for jumping and kicking were investigated through finite element (FE) simulations. The five SLP portions in the FE model were determined by the relative dimensions from the SEM measurements and were defined by the mechanical properties of the fresh SLP cuticles (Table 1). It was simulated that the SLP was distorted proximal-ventrally and the distal-dorsal part of the femur was expanded laterally by 23% during the strain energy storage for jumping. The SLP underwent greater deformation in the strain energy storage process for kicking: the distal tips of the SLPs disappeared behind the dorsal edges of the cover plates, while the sideward expansion of the femoral distal-dorsal part increased up to 33%. The deformations of the fresh distal femur (including the SLP cuticles) are shown in Fig. 6. The reaction force results of the joint pivot were simulated as 27 N and 34 N during the strain energy storages for jumping and kicking, respectively.

**Figure 7:**
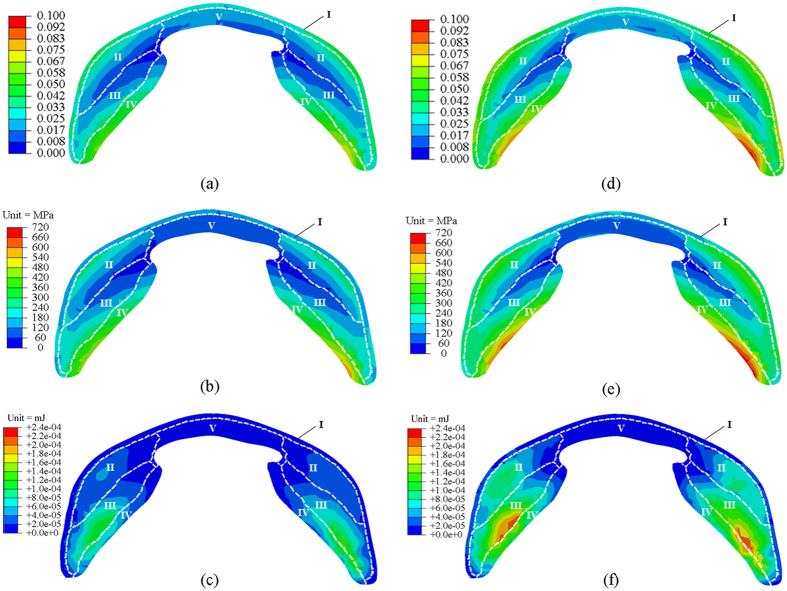
FE simulations of a SLP cuticle during the strain energy storages for jumping and kicking. The distributions of maximal principal strain, von Mises stress, and strain energy in the cross-section of the SLP cuticles are shown in (**a**–**c**) for jumping and in (**d**–**f**) for kicking, respectively. The boundaries of the five SLP portions are marked by white dashed lines. The numbers of the different portions are labeled in all the subfigures. The location of the cross-section is marked by the red line in Fig. 1b. Due to the insignificance of the cover plates in strain energy storage, the distributions in them are not shown here.

All the distributions of maximal principal strain, von Mises stress, and strain energy in the SLP cuticles were compared in Fig. 7. It was found that the highest values of the maximal principal strain all occurred in the endo-ventral part (i.e., portion IV) of the SLP cuticle during strain energy storage (0.07 for jumping and 0.1 for kicking). The exo-surface (i.e., portion I) of the SLP cuticles had some high strain values. Compared with jumping, the maximal principal strains in some partial areas of portions II and III were greatly higher for kicking (Fig. 7a,d). Both the highest von Mises stress and the highest strain appeared in the same portion (portion IV), whilst the von Mises stress in portion II was much higher (270 MPa for jumping and 450 MPa for kicking) than that in portion I (150 MPa for jumping and 210 MPa for kicking). Compared to the results for jumping, the von Mises stresses of the SLP cuticles for kicking had a similar distribution but higher magnitudes (Fig. 7b,e).

The total strain energy storages of the fresh SLP cuticles were simulated to be 4 mJ and 8 mJ for jumping and kicking, respectively. Moreover, the strain energy distribution in the SLP cuticles was distinctly different from those of the maximal principal strain and the von Mises stress (Fig. 7c,f). The highest values of the strain energy all appeared in portion III of the SLP cuticles both for jumping and kicking. In addition, the strain energy values in portion II for kicking was approximately two times higher than those for jumping.

## Discussion

### Microstructural and material characterizations

As shown above, the SLP cuticle of adult *Locusta migratoria manilensis* can be divided into five portions that have different morphologies, microstructures, properties, and functions. On the contrary, the dorsal cuticle of the distal femur mainly has a thin solid shell outside and a multilayered core inside, which is similar to that of the locust sternal cuticles, as observed via histological sectioning and staining^12^. Unlike these cuticles of the dorsal-distal femur, the SLP cuticle is thicker and composed of microfibers in its core, i.e., regularly and irregularly arranged microfibers along the longitudinal direction in portions II and III, respectively (Figs 1c and 2). Portions I and V of the SLP cuticle exhibit similar morphologies to the cuticles on the dorsal-distal femur.

The material compositions of the five SLP portions were analyzed using energy dispersive spectroscopy (EDS), Fourier transform infrared spectrometry (FTIR), and confocal laser scanning microscopy (CLSM). It has been reported that the mechanical properties of some other cuticles are hardened by the presence of mineral elements^15^. Some researchers have found that certain mineral elements (e.g., calcium, magnesium, and sodium) exist in the pincer cuticles of lobsters and crabs, and they suggested that these mineral components account for the mechanical reinforcement of the cuticles^16^,^17^. By contrast, no mineral element has been found in any of the five SLP portions from the EDS analyses. Therefore, the variations in the mechanical properties of the five SLP portions might not be caused by mineral components but by their microstructures, e.g., microfibers.

Although no different functional group is found between the SLP and other cuticles of locusts by FTIR analyses, the CLSM results reveal that the five SLP portions have different proportions of chitin and resilin. Some wide-field fluorescence microscopic images under ultraviolet light have illustrated a great amount of resilin on the inner surface of the SLP^8^, which is in consistency with the blue autofluorescence of our CLSM results. However, the distribution of chitin in the SLP was not determined in their paper. According to our CLSM results, portion III is mainly consisted of chitin, whereas a great amount of chitin is found in portion IV along with a small amount of resilin. Portion II contains a higher proportion of resilin than portions III and IV. Nonetheless, portion II is stiffer than the other two portions, although the elastic modulus of chitin (a few GPa) is much higher than that of resilin (~1 MPa)^10^. The contradiction between the mechanical properties and material compositions of the SLP portions could be explained by their different microstructures (i.e., highly regularly arranged microfibers in portion II). A similar result has been reported by Vincent and its coworkers^15^. They indicate that the cuticles containing 30% chitin and 70% resilin are stiffer than those containing 50% chitin and 50% resilin, which is attributed to the orientation of chitin.

### Mechanical properties

The mechanical properties of the five SLP portions along three orthogonal directions were measured, as shown in Fig. 5. It is found that the mechanical properties of portions I and V are in good agreement with previously reported results for insect cuticles^12^,^18^,^19^. The reduced elastic moduli *E*_*r*_ of the dry tibial exoskeleton of dung beetles along the normal direction are in the range of 3.7–7.6 GPa, whereas their hardnesses *H* are in the range of 270–490 MPa^19^. From the indentation tests of locust sternal plates, the *E*_*r*_ and *H* of the endocuticle along the transverse direction are measured to be 10.5 GPa and 0.3 GPa in the dry condition and 2.7 GPa and 0.04 GPa in the rewetted condition, respectively^12^. These published results of exo- and endo-cuticles are close to our measurements of portions I and V, respectively, except that the portion V of the fresh SLP cuticles has higher stiffness and hardness along the normal direction. In addition, the finding that water reduces both the *E*_*r*_ and *H* values of all the SLP portions is in accordance with published results for some insect cuticles^12^,^18^.

Furthermore, the five portions in the SLP cuticles exhibit greatly different mechanical properties, but they are all approximately transversely isotropic. The anisotropy of portions II and III mainly results from their microstructures consisting of the regularly and irregularly longitudinal aligned microfibers, respectively. The two highest elastic modulus in the fresh SLP cuticles occurs in portions II and III along the longitudinal direction. The mechanical properties of the five portions depend not only on their material compositions but also their microstructures. For example, portion II is composed of both chitin and resilin as regularly arranged microfibers, whereas portion III mainly consists of irregularly arranged chitin microfibers.

### Strain energy-storage mechanisms

The strain energy storage processes for jumping and kicking were simulated using the FE model under the displacement-controlled loading conditions. The simulated deformations of the distal femur, as shown in Fig. 6, coincide with relevant experimental results^1^,^2^. First, our simulations predict a strain energy value of 4 mJ in the SLP cuticles of locusts for jumping, which is the same as that reported by Bennet-Clark^1^. Second, the lateral expansion of the distal femur has also been observed in our study as well as the disappearance of the SLP tip behind the cover plates during the strain energy storage for kicking^2^. However, the stress, strain, and strain energy distributions in the SLP cuticles have not been determined before. Our simulated results reveal that during the strain energy storages for jumping or kicking, both the highest maximal principal strain and von Mises stress appear in the endo-ventral part of the SLP cuticles (i.e., on the endo-surface of portion IV) while the highest strain energy value occurrs in a different region (portion III). Interestingly, a certain amount of resilin is found in portion IV, where high strain and stress have occurred. The resilin plays a toughening role to prevent the occurrence of large plastic deformation or damage in this region^8^.

In comparison with the results for jumping, the total strain energy storage of the SLP cuticle for kicking increases by about two times, whereas the reaction force of the joint only increases by 26%. This may be attributed to the difference in the strain energy distributions in the SLP cuticle. The portion III has higher strain energy values during strain energy storage, whether for jumping or for kicking, whereas the strain energy in portion II becomes high only during the strain energy storage for kicking (Fig. 7f). This finding helps understand the strain energy-storage mechanisms of the SLP cuticles: portion III in the SLP cuticle provides stored strain energy mainly for activities that require less energy (e.g., jumping), and portion II is only involved for activities that require more strain energy (e.g., kicking).

The difference between the strain energy-storage mechanisms of portions II and III is closely related to their microstructures and material compositions. The extension of the locust tibia takes less time for kicking (approximately 6 ms) than for jumping (25–30 ms)^1^,^2^,^3^,^4^,^5^, indicating that a more rapid recovery to its initial shape is required for the deformed SLP during kicking. It has been reported that resilin can help the deformed SLP recover to its original state and release all the stored energy rapidly^8^. The high content of resilin in portion II contributes to enhancing its rapid recovery ability and to satisfying the energy-release demands in such more rapid activities as kicking. Moreover, the microfibers aligned along the longitudinal direction strengthen the mechanical properties of portion II and enhance the energy storage capacity that would be weakened by higher content of flexible tissues (i.e., resilin).

In conclusion, we have revealed the material composition and microstructure of the SLP cuticle in adult *Locusta migratoria manilensis*. It is found that the SLP cuticle has five main parts, which have transversely isotropic properties and heterogeneous distributions of material compositions. With the increase of water content, the mechanical properties of all the SLP portions decrease. FE simulations of the SLP cuticles illustrate that both the highest stress and strain occur on the endo-surface of portion IV (i.e., the endo-ventral part) in the SLP cuticle during the strain energy storages for jumping and kicking. The strain energy is mainly stored by portion III of the SLP cuticle for jumping, but by both portions II and III for activities that require more stored strain energy, e.g., kicking. This research deepens our understanding of the superior strain energy-storage mechanisms of insects such as locusts, which may guide the design and optimization of strain energy-storage devices in bio-inspired robots.

## Materials and Methods

### Specimen preparation

For all the analyses and measurements, mature adult *Locusta migratoria manilensis* of either sex were obtained at least 2 weeks after their final molt from the Jiyuan locust-breeding facility in Anhui Province, China. Thirty-seven locusts were anesthetized, and one hind leg femur (including the SLP) was excised from each insect randomly. All the locust femurs were grouped as follows: one femur was used for measuring the 3D geometry using micro computed tomography (μCT), six femurs were used for microstructural measurements by scanning electron microscopy (SEM) and element analysis by energy dispersive spectroscopy (EDS), six femurs were subjected to compositional analysis using Fourier transform infrared spectrometry (FTIR), six femurs were observed under confocal laser scanning microscopy (CLSM) to determine their material distributions, and eighteen femurs were used in mechanical nanoindentation tests along three orthogonal directions.

### Microstructural observation

The SLP specimens used for the microstructural measurement were cut along their middle cross-section using a razor blade, fixed with 2.5% glutaraldehyde in 0.1 M sodium cacodylate buffer (pH 7.4), dehydrated in a series of ethanol/H_2_O solutions (50%, 70%, 80%, 90% and 100% ethanol), and subjected to critical point drying (Leica EM CPD300, Leica Microsystems GmbH, Wetzlar, Germany). Then, the six samples were mounted on 40-mm SEM mounting plates, gold-palladium sputter coated (Model 682 PECS, Gatan Inc., Pleasanton, CA, USA), and imaged using an SEM with an accelerating voltage of 15 kV (Quanta 200 FEG, FEI Inc., Eindhoven, Netherlands). The whole SLP specimens were viewed at magnifications of 250–500. The microstructure in the cross-section of the SLP was imaged at magnifications of 5000–10,000.

### Elemental and compositional analyses

Using an EDS apparatus mounted in an SEM (EDAX Inc., Mahwah, NJ, USA), the chemical elements in the SLP cuticles were assessed in the same six samples used for the SEM observations. In the compositional analysis, six fresh SLP samples were resected from the locust femur under a biological dissection microscope (XTL-165-VT, Phenix Ltd., Jiangxi, China) and prepared by drying to a powder and mixing with KBr. Then, the SLP samples were tested in a vacuum (vacuum pressure: ~100 Pa) using FTIR (VERTEX 70V, Bruker Inc., Karlsruhe, Germany). In addition, six cuticle specimens from the distal-dorsal part of the same six locust femurs were tested to determine differences between the compositions of the SLP cuticles and the distal-dorsal cuticles of the femur.

All the preparation and measurement methods of CLSM were the same as those in the literature^20^,^21^ and are briefly described as follows. Before the CLSM measurement, six fresh SLP samples were resected along the middle cross-section (indicated by the red line in Fig. 1b) using a razor blade and were immersed in glycerine (≥99.0%; Sinopharm Chemical Reagent Co., Ltd, Shanghai, China). Then, the glycerine-coated SLP cross-sections were placed on a cover slip (thickness = 0.16 ± 0.01 mm; Citotest Labware Manufacturing Co., Ltd, Jiangsu, China) with additional glycerine around them. A CLSM instrument (Zeiss LSM 710META, Carl Zeiss MicroImaging GmbH, Göttingen, Germany) was used for observing the autofluorescences of the specimens under the excitations of 405 nm, 488 nm, 555 nm, and 639 nm laser lines. The transmitting light wavelengths of the emission filter were 420–480 nm (bandpass), ≥490 nm (longpass), ≥560 nm (longpass), and ≥640 nm (longpass), respectively. All the different fluorescences were excited and detected sequentially for avoiding possible interference between the different fluorescences. Laser power values between 3% and 50% were selected, depending on the intensity of the respective autofluorescence. The digital gain and offset values were defined as the default values of the CLSM software ZEN (i.e., 1 and 0, respectively). The pinhole size for each autofluorescence was adjusted slightly by approximately one Airy value to achieve identical thickness of the respective optical sections. The line average of all image stacks was set to 2. According to the rough cross-section of the specimens, the entire thickness of the specimens for the CLSM visualization was set between 100 μm and 200 μm to obtain the whole cross-sectional view of both the SLP and the cover plate. Both the slice thickness and interval values were determined automatically by the ZEN software. Maximum intensity projections were performed for all image stacks using the software. Pseudo-colors of the four different autofluorescences were chosen for the final micrographs as follows: blue (excitation = 405 nm and emission = 420–480 nm), green (excitation = 488 nm and emission ≥490 nm), and red (for both: excitation = 555 nm and emission ≥560 nm, excitation = 639 nm and emission ≥640 nm). To rule out the effect of bleaching on the autofluorescence results, all the micrographs were obtained from the first CLSM visualization procedure of the specimens.

### Mechanical property tests

The SLP specimens for mechanical tests were dried for 24 h, embedded using an EXAKT 520 light polymerization instrument in the light-curing resin Technovit 7200VLC (EXAKT Advanced Technologies GmbH, Norderstedt, Germany), and sectioned with a diamond band saw (EXAKT 300CP, EXAKT Advanced Technologies GmbH, Norderstedt, Germany) along their transverse (plane *Y*-*Z*), tangential (plane *X*-*Z*), and normal (plane *X*-*Y*) cross-sections, as shown in Fig. 1b. All the sections were polished using a grinder-polisher (Metaserv 3000, Buehler Inc., Chicago, Illinois, USA) with polishing suspension (~0.05 microns). The mechanical properties of the SLP cuticles along the longitudinal, tangent, and normal axes were measured using the corresponding sectioned specimens.

First, the sectioned SLP specimens were tested in ambient air using a nanoindentation instrument with a Berkovich diamond indenter (NHTX, CSM Instruments Inc., Switzerland) to obtain the mechanical properties of dry SLP cuticles. The maximum load during indentation was 5 mN, with loading and unloading rates of 10 mN/min. A 10 s hold time at the peak load was included before unloading to minimize the effect of material creep because longer holding times did not induce different results^12^,^22^.

It has been demonstrated that dry locust cuticles soaked in distilled water for 6 h exhibit the same mechanical properties as fresh cuticles^12^,^18^. Therefore, all the dry SLP specimens were rewetted in distilled water for 24 h and tested using a nanomechanical test system (TI 750 ubi, Hysitron Inc., Minneapolis, MN, USA). The mechanical properties of these rewetted SLP samples were considered as those of the fresh SLP specimens in this work. A fluid cell probe with a Berkovich diamond tip (TI-0053, Hysitron Inc., Minneapolis, MN, USA) was employed in the nanoindentation tests because all the tests were performed in distilled water in order to maintain the water content of the samples. The loading settings were the same as those in the tests of the dry SLP samples. The area functions of the two indenters were determined by performing a series of indents at different contact depths on a fused quartz standard sample with the known elastic modulus and hardness.

Both the reduced elastic modulus *E*_*r*_ and the hardness *H* were determined from the indentation data by using the following relations^23^,^24^,^25^:


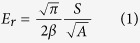



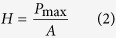


where *P*_max_ is the maximum indentation load, *β* is defined as 1.0207 for the Berkovich tip, and *A* is the area function of the indenter with respect to the contact depth *h*. The contact stiffness *S* was defined as the slope of the initial part of the unloading force-displacement curve and was typically fitted by the upper 20–90% portion of the unloading curve. *E*_*r*_ was determined by


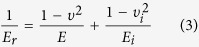


where *E*, υ, *E*_*i*_ and υ_*i*_ are the elastic moduli and Poisson’s ratios of the specimens and the indenter, respectively. Since the diamond tip has a very high elastic modulus (*E*_*i*_ = 1170 GPa), Eq. (3) can be simplified as *E*_*r*_ = *E*/(1−υ^2^).

According to the microstructural images, a SLP cuticle is divided into five main portions. The mechanical properties of the five SLP portions were investigated along three orthogonal directions, namely the longitudinal, tangent, and normal directions. To study the mechanical properties of dry SLP cuticles, 270 indents in the 18 dry SLP specimens (six specimens for each direction) were used, with three indents performed in each portion of each specimen. The mechanical properties of fresh SLP portions were also determined from 270 indents in the 18 rewetted SLP specimens following the same experimental procedure for the dry SLP specimens. The space between adjacent indents always exceeded 20 μm to avoid interference between consecutive measurements. Both the reduced elastic modulus *E*_*r*_ and hardness *H* were calculated from each indentation result by Eqs (1) and (2).

One-way ANOVA (analysis of variance) was performed among the parameters *E*_*r*_ and *H* of the five portions along the same direction to determine the heterogeneity of the properties and among the measurements of the same portion along three different directions to investigate their anisotropy. Bonferroni’s adjustment was applied to all multiple comparisons. A t-test analysis was also performed to compare the indentation properties of the dry and rewetted SLP specimens to determine the effect of water content. A significant difference was defined as a p-value of 0.005 for all tests.

### 3D geometry measurement and FE model reconstruction

The 3D geometry of the locust femorotibial joint was acquired using μCT (Inveon MM Gantry LG, Siemens, Germany). Before scanning, the joint was resected from an adult locust, fixed in Bouin’s fixative for 2 h, dehydrated with graded ethanol solutions (70%, 80%, 90%, and 100%), soaked in 1% iodine/100% ethanol solution at 38 °C for 24 h, and dried in hexamethyldisilazane for 2 h and then in ambient air for 12 h. The specimen was fixed on the μCT stud by adhering the proximal end of the femur onto a PCR tube in the μCT. The specimen was scanned in ambient air at 50 kV and 200 μA with a 2 s exposure time and a resolution of 10.3 μm × 10.3 μm × 10.3 μm voxels.

**Figure 8:**
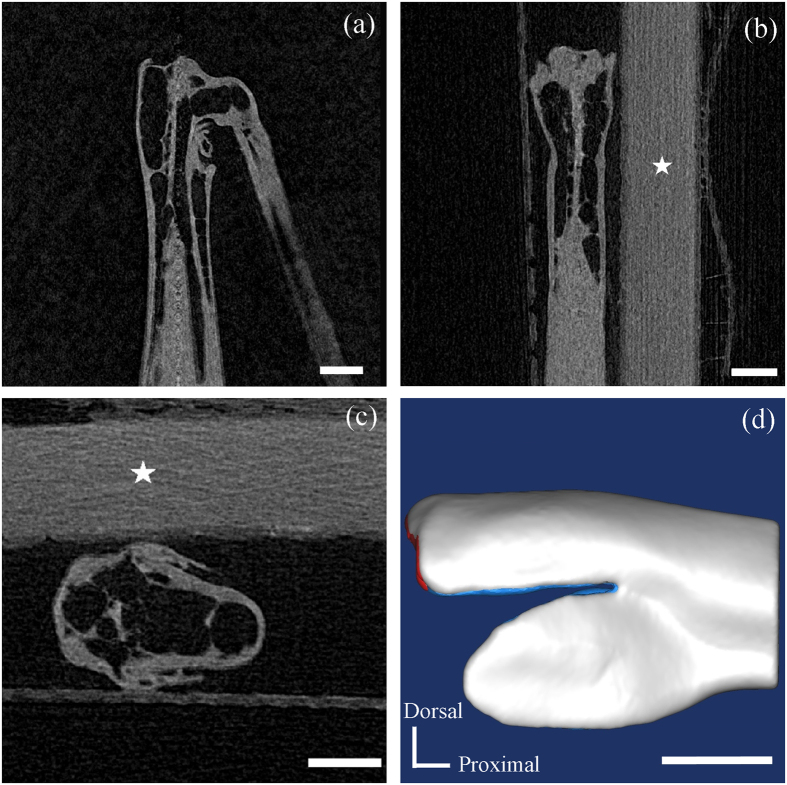
μCT images of the femorotibial joint of the locust and 3D geometric model of its distal femur. (**a**) A 2D μCT slice in the sagittal plane. (**b**) A 2D μCT slice in the coronal plane. (**c**) A 2D μCT slice in the transverse plane. (**d**) Lateral view of the full 3D model of the distal femur. The PCR tube is marked by white stars in (**b**,**c**). Scale bars = 1 mm. An animation of the different portions of the SLP cuticles was presented in the electronic supplementary.

**Figure 9:**
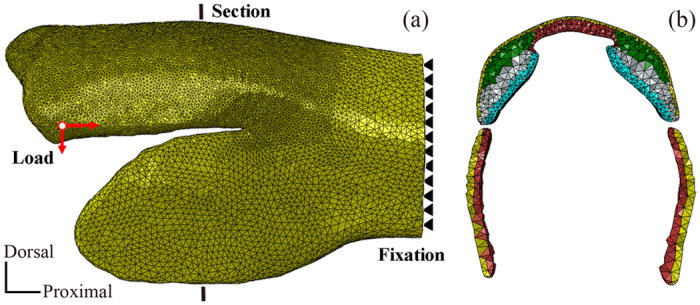
FE model of the distal part of the locust femur. (**a**) The boundary and load conditions in the FE model of the distal femur (in the lateral view) were as follows: The femur was fixed in all DOFs at its proximal end whereas the displacement loads were applied to the pivot of the femorotibial joint (shown by red arrows) to simulate the strain energy storages for jumping and kicking. (**b**) The five portions of the SLP cuticles were simulated by using their corresponding material properties, which are indicated by different colors in the sectional view: I (yellow), II (green), III (gray), IV (blue), and V (red).

A 3D geometrical model of the femur, including the SLP cuticles, was reconstructed from the μCT images using Mimics v13.1 (Materialise Inc., Leuven, Belgium), as shown in Fig. 8 and the animation in the electronic supplementary. The geometrical dimensions of the five different portions of the SLP cuticles were determined based on the relative dimensions of each portion with respect to the overall SLP size, which were obtained by quantitative analysis of the SEM images. Commercial FE software ABAQUS v6.10 (Dassault Systemes Simulia Corp., Providence, RI, USA) was used to simulate the deformation and energy distributions. The 3D geometrical model was meshed into totally 459,684 four-node tetrahedral elements (e.g., element type C3D4). The 3D FE model of the distal femur, including the five portions of the SLP cuticles, is shown in Fig. 9.

In the numerical simulations, all the five SLP portions were assumed to be transversely isotropic. The material coefficients were determined using a published method^26^,^27^,^28^. Refer to a right-handed coordinate system (*x*_1,_ *x*_2_, *x*_3_), where the *x*_3_ axis is normal to the isotropic plane, and the *x*_1_ and *x*_2_ axes are in the isotropic plane. The transversely isotropic elastic constants (i.e., *E*_*P*_, *E*_*T*_, *G*_*P*_, *G*_*T*_, *υ*_*PT*_, *υ*_*TP*_, and *υ*_*P*_) of each portion were calculated from the mean values of the indentation modulus measurements by using the relations:


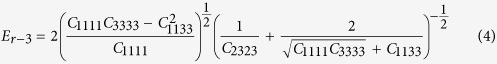



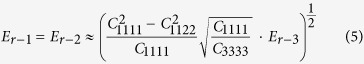


where *E*_*r−1*_ and *E*_*r−2*_ are the reduced elastic moduli in the isotropic *x*_1_ − *x*_2_ plane and *E*_*r−2*_ is the reduced elastic modulus normal to this plane. The relation between the fourth-order stiffness tensor *C*_*ijkl*_ and the transversely isotropic components are


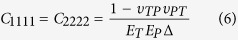



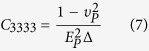



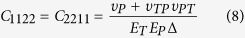



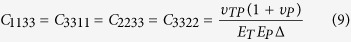



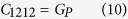



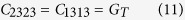


where 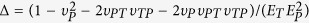. *E*_*P*_ and *E*_*T*_ are the uniaxial Young moduli in the transverse and axial directions, respectively, 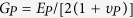 is the in-plane shear modulus. *G*_*T*_ is the out-of-plane shear modulus approximated as 
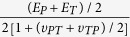
. υ_*PT*_, υ_*TP*_, and υ_*P*_ are the Poisson’s ratios, which have the relations 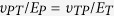 and υ_*PT*_+υ_*TP*_ = 2υ_*P*_^28^. In this work, we set υ_*P*_ = 0.45 for the considered materials. All transversely isotropic coefficients of the five portions of the fresh SLP cuticles were fitted using a custom-written MATLAB script (The MathWorks Corporation, Natick, MA, USA) based on the mean values of nanoindentation measurements, and they were used to define the mechanical properties of the five SLP portions in the FE model (Table 1).

The boundary and load conditions were specified as follows (shown in Fig. 9a). The proximal end of the femur was fixed in all degrees of freedom (DOFs). The frictionless sliding condition was used to simulate the contact pairs by using the general contact algorithm of ABAQUS. According to the previous measurements about the deformation of SLPs^1^,^2^, two displacement loads were applied to the pivot of the femorotibial joint to simulate the strain energy storages for jumping and kicking, corresponding to a combination of 0.2-mm ventral and 0.3-mm proximal movements and a combination of 0.3-mm ventral and 0.4-mm proximal movements, respectively. All the FE simulations were calculated by using ABAQUS v6.10/standard. The distributions of von Mises stress, maximal principal strain, and strain energy in the SLP cuticles were compared.

## Additional Information

**How to cite this article**: Wan, C. *et al.* Structures, properties, and energy-storage mechanisms of the semi-lunar process cuticles in locusts. *Sci. Rep.* **6**, 35219; doi: 10.1038/srep35219 (2016).

## Supplementary Material

Supplementary Video 1

## Figures and Tables

**Table 1:** Transversely isotropic elastic constants of the five SLP portions.

	*E*_*P*_/GPa	*E*_*T*_/GPa	*G*_*P*_/GPa	*G*_*T*_/GPa	υ_*PT*_	υ_*PT*_
Portion I	4.0	2.2	1.4	1.1	0.58	0.32
Portion II	3.2	8.3	1.1	2.0	0.25	0.65
Portion III	5.1	2.1	1.8	1.2	0.64	0.26
Portion IV	2.5	5.2	0.85	1.3	0.29	0.61
Portion V	4.2	1.6	1.5	1.0	0.65	0.25

These values were calculated from the mean values of the nanoindentation tests of 18 fresh SLP samples.
